# Efficacy of increased resistant starch consumption in human type 2 diabetes

**DOI:** 10.1530/EC-14-0036

**Published:** 2014-04-15

**Authors:** C L Bodinham, L Smith, E L Thomas, J D Bell, J R Swann, A Costabile, D Russell-Jones, A M Umpleby, M D Robertson

**Affiliations:** 1 Nutrition, Metabolism and Diabetes Research Group, Faculty of Health and Medical Sciences University of Surrey Leggett Building, Guildford, Surrey, GU2 7WG UK; 2 Metabolic and Molecular Imaging Group MRC Clinical Sciences Centre, Imperial College London UK; 3 Department of Food and Nutritional Sciences University of Reading Whiteknights Campus, Reading, RG6 6AP UK

**Keywords:** euglycemic–hyperinsulinemic clamp, GLP1, flux, stable isotopes

## Abstract

Resistant starch (RS) has been shown to beneficially affect insulin sensitivity in healthy individuals and those with metabolic syndrome, but its effects on human type 2 diabetes (T2DM) are unknown. This study aimed to determine the effects of increased RS consumption on insulin sensitivity and glucose control and changes in postprandial metabolites and body fat in T2DM. Seventeen individuals with well-controlled T2DM (HbA1c 46.6±2 mmol/mol) consumed, in a random order, either 40 g of type 2 RS (HAM-RS2) or a placebo, daily for 12 weeks with a 12-week washout period in between. At the end of each intervention period, participants attended for three metabolic investigations: a two-step euglycemic–hyperinsulinemic clamp combined with an infusion of [6,6-^2^H_2_] glucose, a meal tolerance test (MTT) with arterio-venous sampling across the forearm, and whole-body imaging. HAM-RS2 resulted in significantly lower postprandial glucose concentrations (*P*=0.045) and a trend for greater glucose uptake across the forearm muscle (*P*=0.077); however, there was no effect of HAM-RS2 on hepatic or peripheral insulin sensitivity, or on HbA1c. Fasting non-esterified fatty acid (NEFA) concentrations were significantly lower (*P*=0.004) and NEFA suppression was greater during the clamp with HAM-RS2 (*P*=0.001). Fasting triglyceride (TG) concentrations and soleus intramuscular TG concentrations were significantly higher following the consumption of HAM-RS2 (*P*=0.039 and *P*=0.027 respectively). Although fasting GLP1 concentrations were significantly lower following HAM-RS2 consumption (*P*=0.049), postprandial GLP1 excursions during the MTT were significantly greater (*P*=0.009). HAM-RS2 did not improve tissue insulin sensitivity in well-controlled T2DM, but demonstrated beneficial effects on meal handling, possibly due to higher postprandial GLP1.

## Introduction

It is estimated that 25.8 million children and adults in the USA have diabetes (8.3% of the population), equating to a health cost of $218 billion (∼10% of total healthcare expenditure). Lifestyle interventions, predominantly modulations to dietary intake, are the first-line strategy in diabetes treatment and remain a constant theme throughout management.

Traditionally, dietary fibers have been used to manage blood glucose concentration and have been linked to improved glycemic control in both healthy groups and those with diabetes through various meta-analyses (1). The USDA-recommended fiber intake is 14 g/1000 kcal in healthy individuals, with evidence currently lacking to recommend a higher intake in people with diabetes. This was highlighted in a scientific advisory committee (SACN) statement on nutrition that stated, although cereal fiber intake has been associated with a reduced incidence of type 2 diabetes (T2DM) and metabolic risk factors such as insulin resistance, the majority of evidence relates to T2DM prevention. While this is important given the current obesity epidemic, it cannot necessarily be translated into health benefits for those patients with T2DM (2).

Resistant starch (RS) is a type of cereal fiber and has been shown to have beneficial effects on insulin sensitivity and fatty acid (FA) metabolism in both healthy individuals and those with metabolic syndrome (3, 4, 5, 6, 7). However, the efficacy of RS in individuals with T2DM has not been investigated.

Animal studies have consistently shown that RS improves glucose and insulin metabolism through increased postprandial GLP1 secretion due to stimulation of the colonic enteroendocrine cells (8, 9). This can result in improved insulin secretion. Most recently our own data has shown restored first-phase insulin secretion in metabolic syndrome (10); however, the lack of translational work has recently been highlighted (11). The ADA position on RS states ‘there are no published long-term studies in subjects with diabetes to prove benefits from the use of resistant starch’ (12), whereas the Diabetes Nutrition Study Group of the EASD (13) makes no mention of RS, stating that ‘total dietary fiber should ideally be more than 20 g/1000 kcal’. The current USA and UK fiber intakes equate to ∼7 g and 6 g/1000 kcal, respectively, falling short of even modest guideline daily amounts (GDA).

The primary aim of the study was to translate the beneficial effects of RS feeding reported in healthy and insulin resistant groups, into similar observations in a T2DM cohort. Using an integrated, whole-body approach, stable isotope tracers to differentiate between changes in hepatic and peripheral insulin sensitivity of glucose uptake, body composition analysis using magnetic resonance imaging (MRI) and magnetic resonance spectroscopy (MRS) to investigate changes in body fat depot distribution, and a meal tolerance test (MTT) combined with arterio-venous (A-V) sampling across muscle tissue, for the first time we investigated the efficacy of increasing RS intake to achieve total fiber intakes above the GDA in individuals with T2DM.

## Subjects and methods

### Patients

Seventeen individuals with T2DM (12 males, five females; mean age 55 (s.e.m. 2.4) years, mean BMI 30.6 (s.e.m. 1.3) kg/m^2^) were enrolled in this study. All participants had well-controlled diabetes (mean HbA1c levels of 46.6 (s.e.m. 2) mmol/mol at screening) and were diet and exercise controlled (2/17), taking metformin (13/17) or metformin and pioglitazone (2/17), were weight stable, and excluded if they had a history of gastrointestinal, cardiovascular, or other endocrine diseases.

The study was conducted according to the guidelines laid down in the Declaration of Helsinki, and all procedures were approved by the Kent NHS Research Ethics Committee (10/H1101/29) and the University of Surrey Research Ethics Committee. Written informed consent was obtained from all patients. This trial was registered via the International Standard Randomised Controlled Trial Number 10727538.

### Study design

The study was carried out as a single-blind, randomized dietary intervention crossover study, comparing RS derived from maize with a placebo that was matched for available carbohydrate content. Participants were supplied with supplements labeled either ‘A’ or ‘B’ and were not made aware of their composition. Each supplement was consumed daily for 12 weeks with a 12-week washout period between interventions. During the last week of each intervention period, participants completed a 7-day food and drink diary and a 7-day bowel habit and symptom diary. Participants attended for three study visits at the end of each intervention: i) a two-step euglycemic–hyperinsulinemic clamp combined with an infusion of [6,6-^2^H_2_] glucose, ii) a MTT with A-V sampling across the forearm muscle, and iii) a MRI scan. The first two studies were conducted at the Royal Surrey County Hospital, UK and the third at the MRC Clinical Sciences Center, Hammersmith Hospital, UK. Before each study visit, patients consumed a standard evening meal and then fasted for 12 h. They were instructed to avoid strenuous exercise and alcohol for the preceding 24 h.

Participants were randomized to either 67 g Hi-maize 260 (comprising 60% RS and 40% rapidly digestible starch (RDS) providing 40 g type 2 RS derived from maize, as measured by The Association of Official Analytical Chemists for total dietary fiber method 991.43) or 27 g Amioca (100% RDS). Both supplements were supplied by Ingredion, Inc. (Bridgewater, NJ, USA) in ready-to-use sachets that were mixed into a beverage, and participants were required to consume two sachets daily to obtain the correct dosage. In the results and discussion, the Hi-maize supplement will be referred to as HAM-RS2 and the Amioca supplement as placebo.

#### Euglycemic–hyperinsulinemic clamp with stable isotopes

Participants arrived fasting and following voiding, body weight and composition were measured by bioimpedance (Tanita, Arlington Heights, IL, USA). An i.v. cannula was inserted into each arm for blood sampling and for the glucose, insulin, and isotope infusions. An initial blood sample was collected and a primed continuous infusion of [6,6-^2^H_2_] glucose (170 mg; 1.7 mg/min) commenced. Once a steady state had been reached, five blood samples were taken between 100 and 120 min. At 120 min, the two-step euglycemic–hyperinsulinemic clamp was started. Step one assessed hepatic (endogenous) glucose production (EGP) and involved an insulin infusion (Actrapid, Novo Nordisk, Bagsvaerd, Denmark) of 0.3 mU/kg per min (low dose) for 120 min. Step two assessed insulin sensitivity of glucose uptake (disposal, Rd) and involved an insulin infusion of 1.5 mU/kg per min (high dose) for a further 180 min. Plasma glucose concentrations were maintained at fasting levels through a variable infusion of 20% dextrose spiked with [6,6-^2^H_2_] glucose (8 mg/g for low dose and 10 mg/g for the high dose). Blood samples were taken every 10 min and blood glucose concentrations measured immediately by the glucose oxidase method using the YSI 2300 STAT Plus (YSI Life Sciences, Fleet, UK). Additional samples were taken during the steady state of the low (210–240 min) and high (390–420 min) dose steps.

Owing to difficulties with venous access in some individuals, the data for the euglycemic–hyperinsulinemic clamp are presented for *n*=15 only.

#### MTT with A-V sampling

Following a rest of 10 min, carotid–femoral pulse wave velocity, brachial pulse wave analysis (including measures of mean arterial pressure, aortic pulse pressure, augmentation index, stroke volume, systolic and diastolic aortic blood pressures, and total peripheral resistance), and blood pressure were assessed using the Vicorder system (Smart Medical, Inc., La Mirada, CA, USA).

To assess the metabolism of skeletal muscle *in vivo*, the A-V difference across the forearm muscle was assessed as described previously (5). Arterialized blood was obtained from a cannula inserted into the hand that was placed in a heated box (50 °C). Venous blood was obtained from a cannula placed into deep muscle draining vein of the opposite arm and the hand was occluded for 2 min before drawing each sample using a wrist cuff inflated to 20 mmHg above systolic blood pressure. Oxygen saturation was assessed at each site to ensure correct placement and arterialization, with cutoffs of <60% O_2_ for venous blood and >95% O_2_ for arterialized blood.

Two fasting blood samples were taken simultaneously from both sites. Patients then consumed a standardized liquid meal that did not contain either of the supplements (436 kcal, 61.2 g carbohydrate, 11.9 g fat, and 0 g dietary fiber) at time 0. Further blood samples were collected simultaneously from both sites every half hour for 5 h.

Owing to difficulties with venous access, the postprandial data presented are for 16 patients only and the A-V difference data are for 12 patients only.

#### Magnetic resonance imaging

Upon arrival at the MRI unit, patients underwent whole-body MRI scanning to measure total and regional adipose tissue (AT) contents, as well as liver, pancreatic, and muscle proton MRS (^1^H-MRS) measurements as described previously (3). As not all individuals were suitable candidates for MRI scanning, the data presented are for 14 patients only.

### Biochemistry

Metabolites from the placebo and HAM-RS2 arms of the trial were analyzed together with an intra-assay variation <2.5% for all metabolites.

Plasma samples collected during the clamp were analyzed enzymatically for glucose concentration using a Cobas Mira (Roche Laboratories). Isotopic enrichment of the same plasma samples was measured by gas chromatography–mass spectrometry on an HP5971A mass selective detector (Agilent, Santa Clara, CA, USA). The enrichment was determined using a penta-*O*-trimethylsilyl-d-glucose-*O*-methoxime derivative analyzed by selected ion monitoring of the ions at a charge-to-mass ratio of 319 and 321 (14). Plasma glucose concentrations from the MTT were measured using the glucose oxidase method on the YSI 2300 STAT Plus (YSI Life Sciences) with an inter-assay coefficient of variation (CV) of 1.7%.

Plasma insulin concentrations during the clamp and MTT were measured by ELISA (Millipore, Billerica, MA, USA) with inter-assay CV <14% and intra-assay CV <10%.

Plasma triglyceride (TG), non-esterified FA (NEFA), total cholesterol, and HDL-cholesterol concentrations were measured using commercially available kits for the ILab650 (Instrumentation Laboratory, Warrington, UK), with all inter-assay CV values being <2.5% and all intra-assay CV values being <1.5%.

Fasting tumor necrosis factor α (TNFα) and interleukin 6 (IL6) were measured by commercially available ELISA Kits (2B Scientific, Upper Heyford, UK) with inter-assay CV values <4 and <18% respectively and intra-assay CV values <7 and <10% respectively. Plasma adiponectin and leptin were measured using ELISA (Millipore) with intra-assay CV of 3.6 and 4.4% respectively.

Blood samples for C-peptide and total GLP1 analyses from the MTT were collected into potassium EDTA tubes containing 200 kallikrein inhibiting units (KIU) aprotinin/ml of blood. Plasma samples were then measured by ELISA (Millipore), with inter- and intra-assay CV values <25 and <6.5% for C-peptide and GLP1 respectively.

Serum short-chain FAs (SCFAs) were measured using a gas chromatography-based method as described previously (15, 16). For each sample, 1 μl was injected into a Hewlett Packard 5890 Series II GC system fitted with a Nukol Capillary Column (30 m×0.53 mm×1.0 μm, SUPELCO Analytical, Poole, Dorset, UK) and flame ionization detector. The peaks were integrated using Agilent ChemStation Software, and SCFA contents quantified by single-point internal standard method. Peak identity and internal response factors were determined using a 1 mM calibration cocktail, including acetic, propionic, iso-butyric, butyric, iso-valeric, valeric, ethyl-butyric, and caproic acids.

### A priori and retrospective sample size

Sample size was based on *n*=15 (7). Based on 15 participants completing this crossover study (*α*=0.05), there would be an 82% probability of detecting a treatment difference of 73.7 mmol/l per min in glucose area under the curve (AUC) 0–120 min, based on the assumption of a treatment s.d. of 84, with postprandial glucose tolerance as the primary outcome measure. Both A-V uptake and MRI scanning were not performed due to problems with vascular access and unforeseen claustrophobia in a number of patients. Based on the actual participant numbers, the retrospective power estimate for detecting a difference in the A-V glucose uptake into muscle was 84%, based on a treatment s.d. of 58 and a measured treatment effect of 111 mmol/l per 100 ml tissue. For the MRI measures, the inter-individual variability found in this T2DM cohort was much higher than expected based on data available from those without diabetes. As such, this secondary outcome was significantly underpowered during the study, with only 21% power (*α*=0.05) due to a very high treatment variable s.d. of 4.2.

### Calculations and statistical analyses

LDL-cholesterol was calculated using the Friedewald equation (17). Fasted insulin sensitivity (%S) and β-cell function (%B) were assessed by the homeostatic model assessment (HOMA) (18), and postprandial insulin sensitivity was calculated using the Matsuda index (19).

During the clamp, EGP and glucose Rd were calculated using the model proposed by Steele (20) modified to include the use of stable isotopes. The volume of distribution was assumed to be 22% of body weight. The calculation was also modified for the inclusion of [6,6-^2^H_2_] glucose in the dextrose infusion (21). Before calculation of glucose turnover, plasma glucose concentrations and glucose enrichment time courses were smoothed using optimal segments technique analysis (22). For each time point, EGP and Rd were calculated. Data are expressed as mean EGP and Rd from five sample values collected during each steady state (basal, low, and high dose).

AUC was calculated using the trapezoid rule for 0–120 min (AUC_0–120 min_) for each of the postprandial metabolites. Incremental AUC (iAUC) was also calculated to allow for any significant differences in baseline concentrations. During the MTT, A-V differences in metabolite concentrations were calculated. Total FA uptake into muscle was calculated from the rate of TG and NEFA removal across the tissue as described previously (5).

All dietary analyses were carried out using nutritional analysis software (Dietplan6 Professional version, Forestfield Software, Horsham, UK) and average daily intake was calculated.

All statistical analyses were carried out using SPSS 19.0 for Windows. Statistical significance was taken as *P*<0.05. All data were normalized and analyzed using paired samples *t*-tests and time course data analyzed using repeated-measures ANOVA. Owing to the heterogeneity of the patient group, exploratory Pearson's correlation coefficients were computed to assess potential relationships. For these correlations, percentage change after consumption of HAM-RS2 compared with placebo was calculated. All results are expressed as mean±s.e.m.

## Results

The inclusion of both supplements into habitual diets was well tolerated by the participants as assessed by the bowel habit and symptom diaries. The only reported side effect was a mild but significant increase in the ratings of flatulence with HAM-RS2 compared with placebo (1.1±0.2 vs 0.7±0.1, respectively, *P*=0.006, measured on a scale of 0–4 (none to debilitating)).

There was no significant difference in energy intake between the HAM-RS2 and placebo (2044±116 vs 2089±119 kcal respectively). There was also no significant difference in carbohydrate and fat intakes; however, protein intakes were significantly lower with the HAM-RS2 compared with the placebo (84.2±2.6 vs 92.3±4.8 g, *P*=0.043). As would be expected, mean fiber intake increased significantly during the HAM-RS2 intervention (60.1±1.3 compared with 22.7±1.9 g; *P*<0.001), which can be directly attributed to the HAM-RS2 supplement.

### Glucose metabolism and insulin sensitivity

There was no significant difference, between the HAM-RS2 and the placebo at the end of the 12-week intervention, in fasting glucose or insulin (Table 1) and therefore no significant difference in fasting insulin sensitivity or β-cell function as assessed by HOMA (Table 1). Similarly, there was no significant difference between the interventions in long-term glucose control as assessed by HbA1c (Table 1).

**Figure 1:**
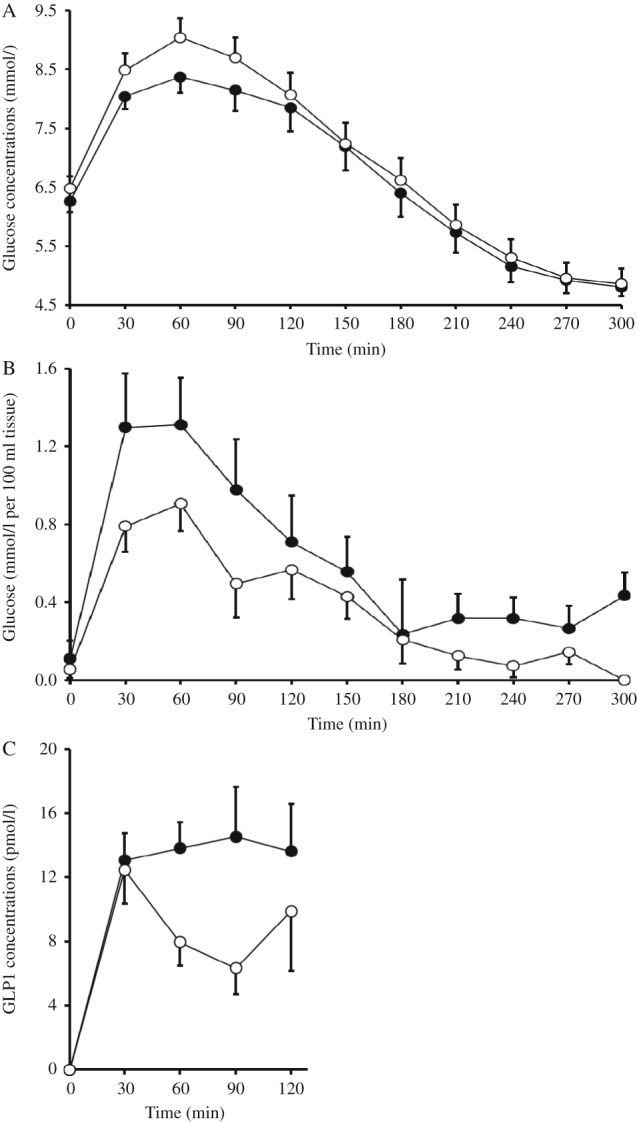
Postprandial glucose concentrations (A), glucose flux into the muscle tissue measured with arterio-venous sampling across the forearm muscle (B), and change from baseline postprandial GLP1 concentrations (C) during the MTT at the end of 12 weeks supplementation with HAM-RS2 (filled circle) compared with placebo (open circle). Mean+s.e.m.s for 16 patients (A and C) and 12 patients (B). There was a significant treatment×time interaction for the postprandial glucose concentrations as assessed by repeated measures ANOVA (*P*=0.045), which corresponded to a significantly reduced AUC_0–120 min_ following the HAM-RS2 supplement (*P*=0.036) compared with paired *t*-test. Repeated measures ANOVA showed a trend for increased glucose uptake with the HAM-RS2 compared with the placebo (*P*=0.077). There was a significantly greater GLP1 response with HAM-RS2 compared with the placebo (*P*=0.009) compared with paired *t*-test on the iAUC_0–120 min_.

During the two-step euglycemic–hyperinsulinemic clamp, no difference was observed between interventions in EGP at basal (placebo, 11.5±0.6 μmol/kg per min and HAM-RS2, 10.7±0.7 μmol/kg per min) or during the low dose insulin infusion (placebo, 4.6±0.3 μmol/kg per min and HAM-RS2, 5.0±0.4 μmol/kg per min). No significant differences were observed between interventions for glucose Rd at basal (placebo, 11.9±0.5 μmol/kg per min and HAM-RS2, 10.7±0.8 μmol/kg per min) or during the high dose insulin infusions (placebo, 47.0±5.4 μmol/kg per min and HAM-RS2, 47.3±6.0 μmol/kg per min). There were no differences in insulin concentrations throughout the clamp study. Postprandial glucose concentrations during the MTT exhibited a significant treatment by time interaction (*P*=0.045; Fig. 1A); this translated as a significant reduction in glucose AUC_0–120 min_ with the HAM-RS2 (*P*=0.036). The A-V sampling across the forearm muscle also showed a trend for higher glucose uptake with HAM-RS2 compared with the placebo (*P*=0.077; Fig. 1B).

The lower postprandial glucose concentrations with HAM-RS2 were not associated with a significant lowering of the plasma insulin concentration; however, there was a significant time×treatment interaction effect on plasma C-peptide concentrations (*P*=0.038; data not shown). There were no significant differences between the interventions for postprandial insulin sensitivity assessed by the Matsuda index (19).

### Fat metabolism

**Figure 2:**
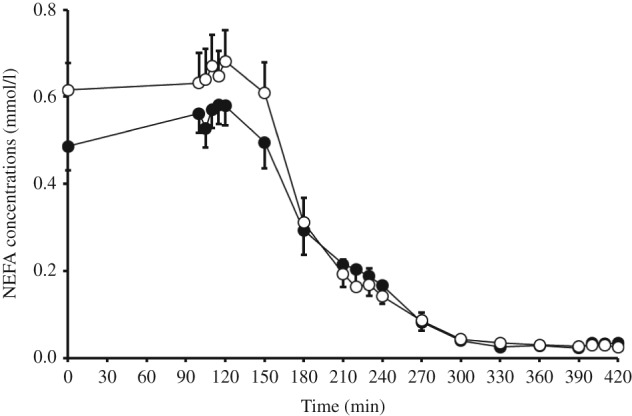
Plasma NEFA concentrations during the two-step euglycemic–hyperinsulinemic clamp at the end of 12 weeks supplementation with HAM-RS2 (filled circle) compared with placebo (open circle). Mean+s.e.m.s for 15 patients. Repeated measures ANOVA showed greater differential suppression of NEFA by insulin with the HAM-RS2 compared with the placebo (*P*=0.001).

Increased consumption of HAM-RS2 resulted in significantly higher fasting TG concentrations (*P*=0.039; Table 1) compared with the placebo, with no effect on cholesterol concentrations. After 12 weeks of increased HAM-RS2 consumption, fasting NEFA concentrations were significantly lower (*P*=0.004; Table 1) and there was greater differential suppression of NEFA by insulin during the two-step clamp (*P*=0.001; Fig. 2). During the MTT, there was no effect of treatment on postprandial concentrations of NEFA or TG and no effect on FA flux as measured by A-V sampling (data not shown); however, the percentage increase in FA uptake into muscle from placebo to HAM-RS2 intake correlated significantly both with an increase in glucose disposal (Rd) measured during the clamp study (*P*=0.036) and with glucose flux measured by A-V sampling in the postprandial state (*P*=0.004).

### Inflammatory markers, SCFA, and hormones

Increased intake of HAM-RS2 for 12 weeks resulted in significantly lower fasting TNFα concentrations (*P*=0.013; Table 1) but had no effects on fasting IL6 concentrations (Table 1). There was also no effect of HAM-RS2 treatment on either fasting plasma leptin or adiponectin concentrations (Table 1). Fasting plasma propionate and butyrate concentrations were significantly lower (*P*=0.021 and *P*<0.001 respectively) following the HAM-RS2 compared with the placebo, but there were no significant differences between treatments for plasma acetate concentrations (Table 1).

Fasting GLP1 concentrations were significantly lower (*P*=0.049) following HAM-RS2 compared with placebo; however, there was a significantly greater meal GLP1 excursion with HAM-RS2 than with the placebo (*P*=0.009; Fig. 1C).

### Vascular function and blood pressure

There were no significant differences in blood pressure, carotid–femoral pulse wave velocity or any of the clinical markers of vascular function (data not shown) following HAM-RS2.

### Body weight and composition

Despite no significant differences in body weight, BMI, or fat mass (Table 1), following 12 weeks of increased HAM-RS2 intake, soleus intramyocellular lipid (S-IMCL) content was significantly higher compared with placebo (*P*=0.027; Table 1); the tibialis IMCL (T-IMCL) was also higher following HAM-RS2 although not significantly (Table 1). This increase in S-IMCL was significantly correlated with the reduction in both fasting NEFA (*P*=0.022) and HbA1c (*P*=0.017). There were no other significant differences between interventions for liver, pancreatic or AT fat depots, assessed by MRS scanning.

## Discussion

Although consumption of HAM-RS2 has been extensively investigated in healthy groups and those with the metabolic syndrome (3, 4, 5, 6, 7, 10), this work represents the first attempt at translation of these findings into an efficacious dietary treatment for human T2DM. In individuals with well-controlled diabetes (mean HbA1c, 46.6 mmol/mol and target UK level for T2DM, 48 mmol/mol), taking oral hypoglycemics, HAM-RS2 intake resulted in a significant improvement in the meal glucose handling (Fig. 1) without a change in medication, habitual diet, exercise, or indeed weight loss. There was, however, no change in the HbA1c following supplementation, but as the intervention was relatively short at 12 weeks in patients already at target HbA1c levels, a longer period may be required for this to become evident.

Although a primary aim of this work was to translate positive results from individuals at increased risk of diabetes into those with the condition (3, 7), it is evident that there are clear differences in the responsiveness between the patient groups to the dietary change. Unexpectedly, intravenous insulin sensitivity as assessed by the hyperinsulinemic clamp technique was not affected by HAM-RS2 intake, despite a positive improvement in oral glucose handling. This disparity between i.v. and oral glucose disposal might imply a gut-mediated factor to be responsible for the effects, a phenomenon often attributed to GLP1. Indeed, GLP1, a well-defined incretin, was found to be elevated postprandially after HAM-RS2 intake, again a finding which was not found in our previous published work in those without diabetes (23) but has been reported in studies of RS in animal models (24). Interestingly, there was no effect of this elevated GLP1 on postprandial insulin levels and so any effect on postprandial glucose disposal may have been through insulin-independent mechanisms. GLP1 has been shown to directly increase muscle glucose uptake in rodent models (25), with the GLP1 receptor recently localized to human skeletal muscle (26). GLP1 acutely raises nitric oxide (NO) levels and so acute changes in both microvascular recruitment (27) and endothelial function (28) at the level of the muscle are believed to be involved in this effect. In the current study, glucose uptake across forearm muscle measured directly using A-V sampling was increased following HAM-RS2 intake and against a background of elevated GLP1 (Fig. 1) although failing to reach statistical significance (*P*=0.077) in the 12 patients in whom arterialized blood samples were obtained. Interestingly, on comparing data obtained from those with T2DM with results obtained using an identical technique in metabolic syndrome patients (7), it appeared that HAM-RS2 intake improved muscle glucose uptake in T2DM patients to levels found in pre-diabetes/metabolic syndrome patients. As HAM-RS2 intake in metabolic syndrome has been shown to normalize glucose uptake to ‘healthy’ levels, it could be hypothesized that a longer period of supplementation would have the potential to improve meal glucose tolerance further in T2DM than achieved during this initial 12-week investigation.

Other metabolic effects attributed to HAM-RS2 intake, such as an improved anti-lipolytic activity of insulin in AT lipolysis, have now also been confirmed in the present study. Both at fasting and under insulin stimulation during the clamp, NEFA concentration was significantly decreased, an effect previously attributed to both stimulation of AT FFA2/3 receptors directly by products of microbial fermentation (29) and to changes at the transcriptional level (7). However, it should be noted that HAM-RS2 consumption did not increase fasting serum SCFA in this study; indeed butyrate and propionate levels were significantly lowered. This may be counterintuitive, but earlier work using the same supplement in healthy individuals also found no impact on fasting SCFA concentrations (7) and, indeed, does not discount a change in fecal/luminal concentrations, which may be involved in the increased stimulation of GLP1 through the FFAR 2/3 receptor. Indeed, recent work using microbial transfer has demonstrated that increasing butyrate-producing bacterial species, for example, does not necessarily result in an increase in butyrate production, implying that the impact of both microbes and dietary fibers is more complex than that of simple SCFA levels (30). An explanation for the lower level of propionate could be the increased clearance into peripheral tissue as demonstrated previously (5) although has yet to be demonstrated *in vivo* in diabetes. Although historically the focus of the reduction in NEFA with fiber feeding has always been in relation to AT content, paradoxically perhaps, a significant increase in S-IMCL was found despite an improvement in glucose tolerance. We have also previously shown this in people with the metabolic syndrome in whom there was a substantial nonsignificant increase (50% increase) in S-IMCL (3). Ectopic fat storage *per se* does not cause insulin resistance (31) and it is hypothesized that the increased partitioning of FAs toward TG storage in a neutral-lipid droplet may be beneficial in blunting the lipotoxicity of lipid species such as ceramides, diacylglycerol (DAG), and fatty acyl coA (32). Indeed, both a single-exercise session (33) and prolonged fasting (34) have been shown to partition more FA toward TG synthesis in skeletal muscle, whereas weight-loss alone does not (35). In this study, the reduction in fasting plasma NEFA correlated significantly with the increase in IMCL in soleus muscle (*P*=0.022). Although the peripheral rate of glucose disposal from the clamp study (Rd) was not significantly different with HAM-RS2 intake, the change in Rd correlated significantly with the change in FA flux into forearm muscle. Combining these lines of evidence suggests that skeletal muscle is a major metabolic target for HAM-RS2 in T2DM, as was found previously in metabolic syndrome. It would be interesting to speculate whether the molecular changes within muscle tissue parallel those found with exercise training such as improved mitochondrial content/function (36).

In accordance with previous data from our group (5, 7), the metabolic impact of HAM-RS2 intake in diabetes would appear to be confined to the periphery (AT and muscle). A new finding in this group of individuals with diabetes was a reduction in obesity-associated inflammation independent from any changes in body fat volume *per se*. Again, as in our previous work in metabolic syndrome (3, 7), our current study found no evidence for an effect on hepatic TG storage or the ability of insulin to inhibit hepatic glucose output (EGP). However, it should be noted that not all the effects observed on lipids could be considered beneficial/neutral. A significant increase in plasma TG was observed in these individuals with T2DM, nonsignificant increases in TG have been observed by our group previously, when RS is used in large doses. In the current study, the increase although statistically significant is unlikely to be clinically significant as participants had TG levels within the reference range. However, the implication for individuals with baseline hyperlipidemia is unknown and warrants investigation.

In conclusion, this is the first RS feeding study in human T2DM where the metabolic effects of RS (rather than a manipulation of dietary glycemic index/glycemic load (37)) have been investigated. HAM-RS2 intake improved meal glucose tolerance in patients with existing good diabetic-control due to a mechanism which appears to involve increased muscle uptake of FAs and increased S-IMCL. However, as a caveat, changes in both ectopic TG distribution and plasma TG were found, the clinical significance of which is unknown. Further work is now warranted to elucidate the molecular mechanisms within muscle tissue attributable to HAM-RS2, which would be vital in terms of recommending diet/exercise interventions to maximize the benefits for muscle glucose uptake. A larger scale intervention should now be undertaken in patients using high-fiber foods, with less well-controlled diabetes and over a longer time frame before a change to the evidenced-based dietary guidelines could be proposed.

## Author contribution statement

C L Bodinham conducted the clinical experiments, analyzed the data, and wrote the manuscript; L Smith assisted with the conduction of clinical experiments, analysis of data, and edited the manuscript; E L Thomas acquired and analyzed the data and edited the manuscript, J D Bell edited the manuscript; J R Swann acquired and analyzed the data and edited the manuscript; A Costabile acquired and analyzed the data; D Russell-Jones supervised the clinical work; A M Umpleby analyzed the data and edited the manuscript; M D Robertson assisted with the conduction of clinical experiments, analyzed the data, and wrote the manuscript. M D Robertson is the guarantor of the work and had full access to all of the data in the study and takes responsibility for the integrity of the data and the accuracy of the data analysis. Parts of this study were presented in abstract form at the 73rd Scientific Sessions of the American Diabetes Association, Chicago, June 2013.

## Figures and Tables

**Table 1:** Anthropometric measurements, body fat depots, and fasting plasma concentrations taken after 12 weeks supplementation with 40 g/day HAM-RS2 compared with placebo. Mean±s.e.m. for 17 patients.

	**HAM-RS2**	**Placebo**	***P* value**
Body weight (kg)	92.5±5.0	91.7±5.1	NS
BMI (kg/m^2^)	31.0±1.3	30.7±1.4	NS
Fat mass (kg)^a^	32.2±2.7	31.8±2.9	NS
Total AT (l)^b^	35.8±3.6	34.5±4.0	NS
Subcutaneous AT (l)^b^	26.2±2.9	25.6±3.3	NS
Internal AT (l)^b^	9.6±1.1	9.0±1.0	NS
IHCL^b^	9.7±2.7	10.0±3.3	NS
Pancreas fat^b^	13.7±3.9	10.5±3.2	NS
S-IMCL^b^	24.7±4.6	19.3±3.7	0.027
T-IMCL^b^	7.9±1.1	6.5±0.8	NS
HbA1c (mmol/mol)	46.8±1.5	47.9±2.0	NS
Glucose (mmol/l)	6.2±0.2	6.4±0.2	NS
Insulin (pmol/l)	49.1±7.4	51.0±9.5	NS
C-peptide (nmol/l)	0.7±0.1	0.7±0.1	NS
HOMA %S	116.3±15.1	115.9±13.9	NS
HOMA %B	67.5±7.1	63.5±6.8	NS
NEFA (μmol/l)	500±100	600±50	0.004
TG (mmol/l)	1.4±0.1	1.2±0.1	0.039
Total cholesterol (mmol/l)	3.6±0.1	3.4±0.2	NS
HDL-cholesterol (mmol/l)	1.0±0.1	1.0±0.1	NS
LDL-cholesterol (mmol/l)	1.9±0.1	1.8±0.1	NS
GLP1 (pmol/l)	11.4±1.9	17.0±3.2	0.049
Leptin (ng/ml)	10.9±1.9	10.2±1.8	NS
Adiponectin (ng/ml)	8232±1249	7701±879	NS
TNFα (pg/ml)	5.1±2.6	13.2±3.9	0.013
IL6 (pg/ml)	6.5±1.8	3.8±1.5	NS
Acetate (μmol/l)	99.1±1.2	98.3±6.1	NS
Propionate (μmol/l)	4.0±0.2	7.5±1.4	0.021
Butyrate (μmol/l)	0.6±0.1	1.1±0.1	<0.001

AT, adipose tissue; IHCL, intrahepatocellular lipid; S-IMCL, soleus intramyocellular lipid; T-IMCL, tibialis IMCL; HOMA %S, fasted oral insulin sensitivity assessed by homeostasis model assessment; HOMA %B, β-cell function, assessed by HOMA (18).

a Measured by bioimpedance (*n*=16).

b Body fat depots determined by MRS scanning (*n*=14).
